# Chemokine and Cytokine Cascade Caused by Skewing of the Th1-Th2 Balance Is Associated with High Intracranial Pressure in HIV-Associated Cryptococcal Meningitis

**DOI:** 10.1155/2019/2053958

**Published:** 2019-12-31

**Authors:** Lijun Xu, Yongzheng Guo, Yizhou Zhao, Yufan Xu, Xiuming Peng, Zongxing Yang, Ran Tao, Ying Huang, Yan Xu, Yaokai Chen, Biao Zhu

**Affiliations:** ^1^National Clinical Research Center for Infectious Diseases, The First Affiliated Hospital, College of Medicine, Zhejiang University, Qingchun Rd, Hangzhou, China; ^2^The State Key Laboratory for Diagnosis and Treatment of Infectious Diseases, The First Affiliated Hospital, College of Medicine, Zhejiang University, Qingchun Rd, Hangzhou, China; ^3^College of Medicine, Zhejiang University, Yuhangtang Rd, Hangzhou, China; ^4^Department of HIV/AIDS, Xixi Hospital of Hangzhou, Hengbu Rd, Hangzhou, China; ^5^Chongqing Public Health Medical Center, Baoyu Rd, Chongqing, China

## Abstract

**Purpose:**

Serum cytokines/chemokines play important roles in cryptococcal meningitis, but it is unclear whether cytokines/chemokines in cerebrospinal fluid (CSF) contribute to high intracranial pressure (HICP) in HIV-associated cryptococcal meningitis (HCM).

**Methods:**

CSF cytokines/chemokines were assayed in 17 HIV-uninfected patients, 26 HIV-infected patients without CNS infection, and 39 HCM patients at admission. Principal component analysis and correlation and logistic regression analyses were used to assess the relationships between these parameters.

**Results:**

The CSF Th1, Th2, and macrophage cytokines showed an obvious increase in HCM patients as compared to the HIV-uninfected patients and HIV-infected patients without CNS infection. CSF IL-6, GM-CSF, and IL-8 were positively correlated with CSF fungal burden. Serum CD4 count, CSF Th1 cytokines (TNF-*α*, TNF-*β*, IL-12, IL-1*β*, IL-12, IL-1*α*, TNF-*α*, TNF-*β*, IL-12, IL-1*γ*, and IL-12) and Th2 cytokines (IL-4 and IL-10) contribute to HICP.

**Conclusion:**

Overall, the present findings indicated that both pro- and anti-inflammatory cytokines of Th1, Th2, and macrophage origin contributed to the development of HCM. Specifically, the chemokine and cytokine cascade caused by skewing of the Th1-Th2 balance and reduced CD4 count were found to be important contributors to HICP. *Summary*. Our research suggested that chemokine and cytokine cascade caused by skewing of the Th1-Th2 balance in HIV-infected patients played more important role than Cryptococcus numbers and size in CSF on the development of high intracranial pressure in HIV-associated cryptococcal meningitis, providing a new understanding of mechanisms of HCM.

## 1. Introduction

HIV-associated cryptococcal meningitis (HCM) is a leading cause of mortality (with a mortality rate of 30-50%) in AIDS patients, especially in patients in low-resource settings [1, 2]. High intracranial pressure (HICP) is one of the important risk factors for mortality in HCM patients [3, 4]. HICP may present as the primary clinical manifestation in untreated HCM patients and also in patients who have undergone antifungal therapy for a period of time [3, 5]. Therefore, HICP is considered as a paradoxical clinical manifestation in HCM patients. It is believed that one of the causes of HICP is HIV-associated immune reconstitution inflammatory syndrome (IRIS), which is characterized by an exaggerated inflammatory response in a subset of patients occurred after the initiation of antiretroviral therapy (ART) [6, 7]. However, the mechanisms underlying the development of HICP are complicated and debatable; hence, further investigation is required to clarify the mechanisms.

Previous studies indicate that larger Cryptococcus capsule size, higher fungal burden, and higher cryptococcal antigen titers in CSF are associated with HICP [4, 5, 8]. However, HICP may also be present in some patients with low fungal burden [9]. In addition, HICP may continue to persist, even after the cryptococcal titer has decreased in response to antifungal treatment [10]. In particular, HICP may present after the initiation of ART, even after a few weeks of anticryptococcal treatment. From these contradictory findings, it can be deduced that the fungal load or cryptococcal antigen titers in CSF alone are not sufficient to explain the HICP in HCM patients. Therefore, it is important to explore other clinical parameters that may be associated with the development of HICP in these patients.

Serum cytokines and/or chemokines have emerged as important players in the development of HICP in HCM patients in recent years, but the related findings are inconsistent. For example, a study by Boulware et al. indicated that high serum pre-ART levels of IL-4 and IL-17 are predictive of future IRIS in HCM patients [11], whereas Zheng's research indicated that decreased IL-4 and IL-17 levels were associated with HICP during IRIS [12]. These paradoxical research findings indicate the need to investigate further the roles of cytokines/chemokines in HICP. Importantly, simple serum measurements of these cytokines/chemokines may not be sufficient, because the CSF levels do not always correspond with the serum levels of the same cytokines in some diseases [13, 14]. In fact, local factors might play an important role on the CNS disease. Thus, assay of CSF cytokines/chemokines, instead, might provide a better understanding of the mechanism of HICP. In fact, a study by Jarvis et al. reported that higher CSF levels of IL-6, IL-8, IL-10, and IFN-*γ* were indicative of the risk of HICP in patients with IRIS [15], and this formed the basis of the present study.

We explored the mechanism of HICP in HCM patients by assessing their CSF cytokine/chemokine profile.

## 2. Methods

### 2.1. Study Population and Diagnosis

The study sample included 17 HIV-negative patients (5 patients had been initially suspected of having CNS infection that was later ruled out, and 12 were leukemia patients without CNS involvement who agreed to undergo routine CSF examination to exclude invasive CNS leukemia), 26 HIV-infected patients without CNS infection (who agreed to undergo lumbar puncture for suspected CNS infection), and 39 patients with HCM. CM was diagnosed if the patient met at least one of these criteria: (i) positive CSF India ink smear test, (ii) positive Cryptococcus CSF culture, or (iii) positive CSF cryptococcal antigen. All the patients agreed to undergo lumbar puncture during their first week of admission to the hospital. All the HCM patients were treated with amphotericin B and fluorocytosine, with or without fluconazole [16]. Pretreatment CSF samples were collected between Jan 2013 and Dec 2016 and frozen at -80°C for subsequent analysis.

HICP was defined by an open CSF pressure of ≥300 mmH_2_O. Patient death or agreement to undergo ventriculoperitoneal shunt placement was considered as adverse outcomes.

### 2.2. Laboratory Tests

Blood samples were drawn after a 12 h fast within 3 days after the patient was admitted. The CD4+ and CD8+ T cell numbers were determined using flow cytometry (B&D Science, NJ, USA) with FITC-conjugated anti-human CD4, PE-conjugated anti-human CD8, and PE-Cy5-conjugated anti-human CD3 mAbs (B&D Sciences, NJ, USA).

According to our pretest results, the following cytokines/chemokines were assayed in the CSF samples of all the patients: IL-1*β*, IL-1R*α*, IL-4, IL-5, IL-6, IL-8 (chemokine (C-X-C motif) ligand 8), IL-10, IL-12p40, IL-12p70, CD40L, IFN-*γ*, IFN-*α*2, TNF-*α*, TNF-*β*, RANTES (chemokine (C-C motif) ligand 5), granulocyte-macrophage colony-stimulating factor (GM-CSF), and monocyte chemotactic protein-1 (MCP1). The Luminex multianalyte platform (Luminex) and MILLIPLEX® map (Cat. No. HCYTMAG-60K-PX38) were used for the assays.

The procedure of Cryptococcus count was briefly described: 1 ml of CSF was collected and centrifuged at the speed of 3000 rpm × 10 − 15 min, and then, the sediment (≈100 *μ*l) was mixed with a small drop of India ink. A large coverslip was applied over 100 *μ*l of mixture on the glass slide and pressed gently to obtain a thin mount. The slide was scanned under low-power field in reduced light with a microscope and then switched to high-power field (HPF) for counting if cryptococcal capsules were found. The Cryptococcus count (cells/HPF) = (total number of Cryptococcus capsules in 10 HPFs)/10 [17].

### 2.3. Statistical Analysis

Continuous normal variables are expressed as mean ± standard deviation, and categorical variables are expressed as the number of cases (percentage). The CD4+ T cell counts are expressed as median (interquartile range (IQR)). The chemokine (ng/ml) and cytokine (ng/ml) concentrations were log_10_-transformed for statistical analysis. Continuous normal variables were compared by one-way ANOVA (LSD method) or Student's *t*-test, and continuous abnormal variables were compared by nonparametric tests (Mann–Whitney *U* or Kruskal-Wallis *H* test). Categorical variables were compared by *χ*^2^ analysis or Fisher's exact test. The association of CSF cytokine/chemokine levels with HCM and HICP was analyzed by principal component analysis (PCA). Associations between the principal components (PCs) and CD4 count, CSF lymphocyte count, baseline fungal burden, HCM acquisition, HICP, and adverse outcomes were examined using Spearman/Pearson's correlation analysis or logistic regression. *P* values < 0.05 (two-tailed) were considered to indicate statistical significance. Data analysis was performed using the SPSS 24.0 statistical software (SPSS Inc., IL, USA) and GraphPad 7 (GraphPad Software, California, USA).

### 2.4. Ethics Statement

The study protocol was in accordance with the 1975 Declaration of Helsinki and approved by the Ethics Committee of the First Affiliated Hospital, School of Medicine, Zhejiang University (Hangzhou, China) (No. 2017-688). All human subjects were adult, and written informed consent was obtained from all the patients prior to their participation in the study.

## 3. Results

### 3.1. Patient Characteristics

Our study cohort included 70 (85.4%) male and 12 (14.6%) female patients, including 17 HIV-negative patients and 65 ART-naïve HIV-infected patients. Of those HIV-infected patients, there were 26 HIV patients without CNS infection and 39 HCM patients. The mean age of the patients was 36.9 ± 11.5 years. Most HIV-infected patients had advanced immunosuppression status with a CD4 cell count of 38 (17–89) cells/*μ*l. The HIV patients without CNS infection had a CD4 cell count of 82 (18-266) cells/*μ*l, and the HCM patients had a CD4 cell count of 35 (15-59) cells/*μ*l (*P* = 0.043). The median ICP of all the patients was 190 (140-350) mmH_2_O among the 39 HCM patients. 19 HCM patients had ICP < 300 mmH_2_O, and 20 HCM patients had ICP ≥ 300 mmH_2_O. Seven (7/39, 11.3%) patients whose ICP above 300 mmH_2_O (which is indicative of HICP) underwent ventriculoperitoneal shunt operation for the reduction of HICP, and 2 (2/39, 5.1%) patients died during 10-week follow-up. The baseline demographic and clinical characteristics of the patients are shown in Table 1.

## 4. Association of ICP with the CD4 Cell Count in the Peripheral Blood of HCM Patients

**Figure 1:**
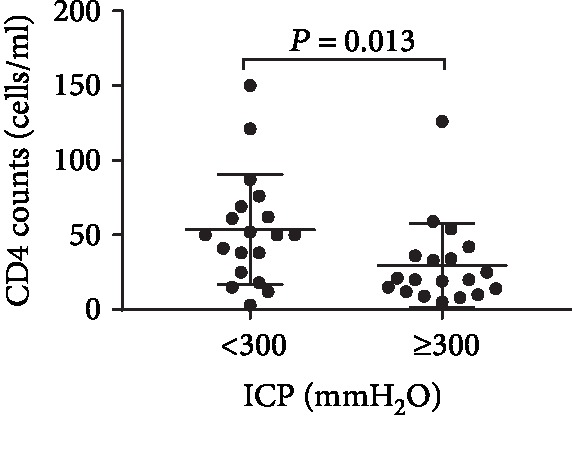
CD4 count and open intracranial pressure in HCM patients. Patients with ICP ≥ 300 mmH_2_O had a lower CD4 count than those with ICP < 300 mmH_2_O (*P* = 0.013).

To analyze the relationship between HIV/AIDS status and ICP, the patients were subgrouped into those with ICP < 300 mmH_2_O and those with ICP ≥ 300 mmH_2_O. The peripheral CD4 cell count was 50 (25-69) cells/*μ*l in patients with ICP < 300 mmH_2_O and 20 (12-36) cells/*μ*l in patients with ICP ≥ 300 mmH_2_O (*P* = 0.013) (Figure 1). Correlation analysis showed that the CD4 cell count was negatively associated with ICP (*r* = ‐0.349, *P* = 0.032). Additionally, the levels of chlorine, glucose, total protein, albumin, and *β*2-microglobulin in CSF were analyzed, but they were not significantly different between the two ICP subgroups (*P* = 0.197, *P* = 0.473, *P* = 0.447, *P* = 0.792, and *P* = 0.430, respectively).

## 5. Differences in CSF Cytokine and Chemokine Levels between the Patient Groups

IL-1*β*, IL-1R*α*, IL-4, IL-5, IL-6, IL-8, IL-10, IL-12p40, IL-12p70, CD40L, INF-*α*2, INF-*γ*, TNF-*α*, TNF-*β*, RANTES, GM-CSF, and MCP-1 were assayed in all the patients. The test results indicated that were no differences in the CSF levels of all the proinflammatory cytokines (IL-1*β*, IL-8, IL-10, IL-12p40, GM-CSF, RANTES, TNF-*α*, INF-*γ*, MCP-1, and CD40L) between the HIV-negative patients and HIV patients without CNS infection. In contrast, the CSF levels of all the proinflammatory cytokines, with the exception of GM-CSF, were obviously higher in the HCM patients than in the HIV patients without CNS infection or in the HIV-negative patients. With regard to the anti-inflammatory cytokines, their CSF levels were similar in the HIV patients without CNS infection and the HIV-negative patients; however, the levels of IL-1R*α*, IL-4, IL-6, IL-10, and INF-*α*2 in the HCM group were significantly higher than those in the other two groups (*P* < 0.001 for all). Finally, the TNF-*β* level was significantly higher in the HCM patients than in the other two groups of patients (*P* < 0.05).

**Figure 2:**
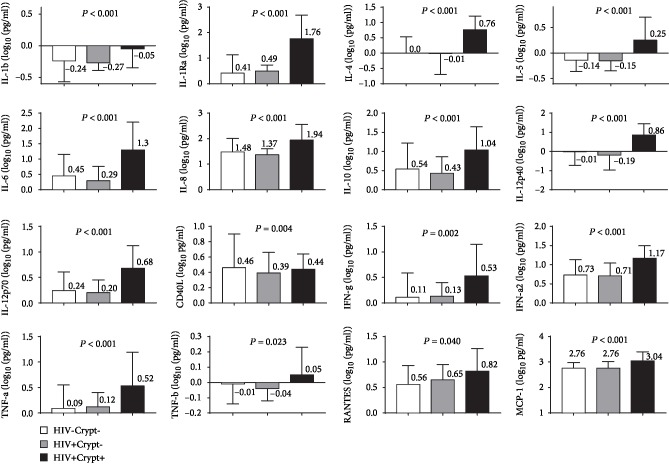
Baseline cytokine and chemokine levels in HIV-uninfected patients (HIV-Crypt-), HIV-infected patients (HIV+Crypt-), and HIV-infected patients with cryptococcal meningitis (HIV+Crypt+). Most baseline cytokine and chemokine levels were significantly elevated in HIV+Crypt+ patients (GSF-GM levels were not shown because no significant difference was found between groups).

With regard to the origin of the cytokines and chemokines, we found that the levels of the Th1 cytokines IL-10, IL-12, TNF-*α*, and TNF-*β* were significantly higher in the HCM patients than in the HIV patients without CNS infection and in the HIV-negative patients (*P* < 0.05 for all). Similarly, the levels of the Th2 cytokines IL-4, IL-5, IL-6, and IL-10 were significantly higher in the HCM patients than in the other two groups of patients (*P* < 0.01 for all) (Figure 2).

## 6. Identification of Cytokines and Chemokines Associated with HCM

**Figure 3:**
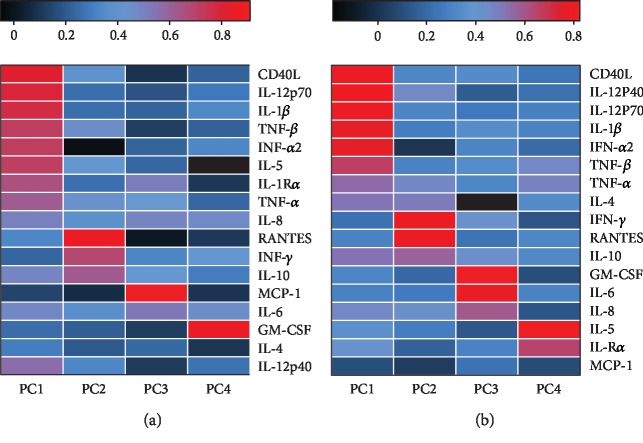
Principal component analysis scores and weightings in a heat map. (a) Variance of the 4 principal component scores among the 65 HIV-infected patients. PC1 and PC3 were correlated with HIV-associated cryptococcal meningitis (HCM). PC1 was composed primarily of proinflammatory cytokines such as CD40L, IL-1*β*, IL-8, IL-12, and TNF-*α* and anti-inflammatory cytokines such as IFN-*α*2, TNF-*β*, and IL-8. PC3 was majorly driven by MCP-1, IL-6, and IL-R*α*. (b) Proportion of variance in the 4 principal components in the 39 HCM patients. PC1 was correlated with high intracranial pressure (HICP). Component loading indicated that CD40L, IL-12, IL-1*β*, IFN-*α*2, TNF-*α*, TNF-*β*, IL-4, and IL-10 were the main components of PC1.

PCA was used to assess the association of the assayed cytokines and chemokines with HCM. The majority of the variance was reflected by PC1 (66.2%), PC2 (6.1%), PC3 (5.6%), PC4 (4.4%), and PC5 (3.6%) in all the patients. As depicted by the data, 82.3% of the total variance was reflected by PC1, PC2, PC3, and PC4. Therefore, PC1, PC2, PC3, and PC4 were used for further analysis. Logistic regression analysis indicated that PC1 and PC3 were associated with Cryptococcus infection in the CNS, but PC2 and PC4 were not. The hazard ratios were 2.460 (95% confidential interval (CI): 1.566-3.889) for patients with higher PC1 (*P* < 0.001) and 0.229 (95% CI: 0.081-0.644) for patients with PC3 (*P* = 0.005). The component loadings for each variable showed that the variance in PC1 was driven by positive loading scores for proinflammatory cytokines, such as CD40L, IL-1*β*, IL-8, IL-12, and TNF-*α*; further, anti-inflammatory cytokines, such as IFN-*α*2, TNF-*β*, and IL-8, also made positive contributions to the PC1 score. Finally, PC3 was majorly driven by MCP-1, IL-6, and IL-8 (Figure 3(a)). These findings indicate that the pathogenesis of HCM involves both proinflammatory and anti-inflammatory cytokines.

## 7. Identification of Cytokines and Chemokines Associated with HICP and Adverse Outcomes in HCM Patients

We identified five other PCs to assess variance in the cytokine/chemokine levels in the 39 HCM patients. PC1 was responsible for 65.2% variance; PC2, for 7.4%; PC3, for 5.9%; PC4, for 4.9%; and PC5, for 3.5%. Thus, PC1, PC2, PC3, and PC4 reflected 83.3% of the variance. Spearman's correlation analysis indicated that PC1 was significantly correlated with the CSF lymphocyte count (*r* = 0.668, *P* < 0.001) and ICP (*r* = 0.354, *P* = 0.027); PC3 was significantly correlated with the CSF Cryptococcus count (*r* = 0.471, *P* = 0.003) and negatively correlated with the CSF lymphocyte count (*r* = ‐0.354, *P* = 0.037); and PC4 was positively correlated with the CD4 cell count in peripheral blood (*r* = 0.420, *P* = 0.009). Linear regression was used to analyze the relationships between CSF *Cryptococcus* count and PC1, PC2, PC3, PC4, CD4 cell count in peripheral blood, and CSF lymphocyte count. Our data suggested that only PC3 was tightly close to higher CSF *Cryptococcus* (6.972 (95% CI: 2.471-11.473), *P* = 0.004). Component loading indicated that PC3 was positively driven by GM-CSF, IL-6, and IL-8 and was negatively driven by IL-4.

Among the 39 HCM patients, 19 had ICP < 300 mmH_2_O and 20 had ICP ≥ 300 mmH_2_O. Logistic regression was used to analyze the factors that potentially influence ICP, including the CD4 cell count in peripheral blood, CSF Cryptococcus count, and CSF lymphocyte count, as they were found to be significantly correlated with PC1, PC2, PC3, and PC4. The results indicated that the hazard ratio for ICP ≥ 300 mmH_2_O was 3.490 (95% CI: 1.456-8.361) for patients with higher PC1 (*P* = 0.005), 0.954 (95% CI: 0.922-0.988) for patients with higher CSF lymphocyte count (*P* = 0.009), and 0.912 (95% CI: 0.852-0.977) for patients with higher CD4 count (*P* = 0.008). In contrast, the CSF Cryptococcus count (*P* = 0.949), PC2 (*P* = 0.731), PC3 (*P* = 0.402), and PC4 (*P* = 0.056) did not emerge as risk factors for the development of ICP ≥ 300 mmH_2_O.

**Figure 4:**
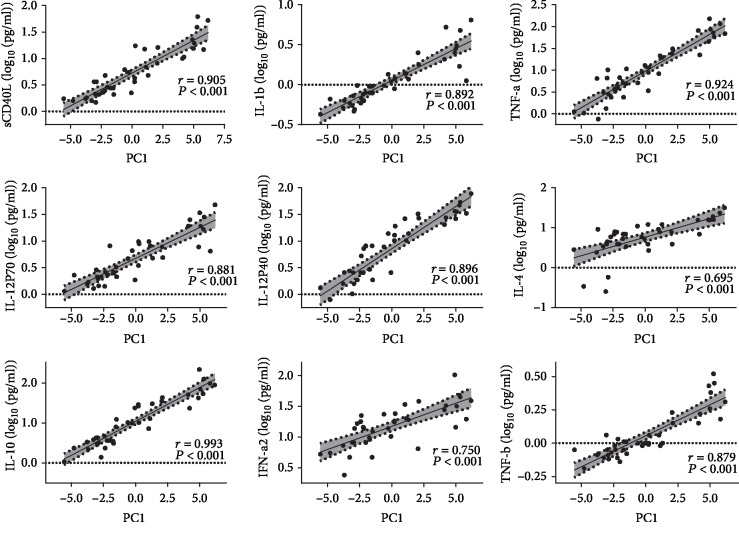
Relationship between cytokine/chemokine concentrations and PC1 in the 39 HCM patients. Associations between baseline proinflammatory or anti-inflammatory cytokine concentrations and PC1 were analyzed with best-fit regression lines with 95% confidence intervals.

Component loading indicated that PC1 was driven by CD40L, IL-12, IL-1*β*, IFN-*α*2, TNF-*α*, TNF-*β*, IL-4, and IL-10 (Figure 3(b)). Further, Pearson's correlation analysis and best-fit regression indicated that PC1 was associated with the proinflammatory cytokines IL-1*β* (*r* = 0.892, *P* < 0.001), TNF-*α* (*r* = 0.924, *P* < 0.001), IL-12p40 (*r* = 0.896, *P* < 0.001), IL-12p70 (*r* = 0.881, *P* < 0.001), and CD40L (*r* = 0.905, *P* < 0.001) and the anti-inflammatory cytokines IL-4 (*r* = 0.695, *P* < 0.001), IL-10 (*r* = 0.933, *P* < 0.001), IFN-*α*2 (*r* = 0.750, *P* < 0.001), and TNF-*β* (*r* = 0.879, *P* < 0.001) (Figure 4). This finding indicates that both proinflammatory and anti-inflammatory cytokines/chemokines contribute to HICP.

Adverse outcomes occurred in 9 cases of 39 HCM patients: 2 patients died and 7 patients underwent ventriculoperitoneal shunt operation during the 10-week follow-up. CD4 count, CSF fungal burden, CSF lymphocyte count, PC1, PC2, PC3, PC4, and ICP were used for logistic regression analysis. Our data indicated that only CSF fungal burden was a risk factor for adverse outcomes (hazard ratio: 1.4 (95% CI: 1.0-1.8), *P* = 0.032), whereas PC1 (*P* = 0.071), CSF lymphocyte count (*P* = 0.161), PC2 (*P* = 0.887), PC3 (*P* = 0.136), PC4 (*P* = 0.669), and CD4 (*P* = 0.540) were not significantly associated with adverse clinical outcomes.

## 8. Discussion

In the present study, we have examined the level of 17 chemokines/cytokines in the CSF samples of 82 patients, including 17 patients without HIV infection, 26 patients with HIV but no CNS infection, and 39 HIV patients with CM or HCM patients (19 with ICP < 300 mmH_2_O and 20 with ICP ≥ 300 mmH_2_O), in order to investigate the associations between cytokines/chemokines in CSF and HICP in HCM patients. Our data indicate that (1) lower CD4 count in peripheral blood and lower CSF lymphocyte count are both associated with HICP; (2) the pretreatment levels of the chemokine/cytokines CD40L, IL-12, IL-1*β*, INF-*α*2, TNF-*α*, and TNF-*β* are closely associated with Cryptococcus infection and HICP in HCM patients (additionally, the IL-5, IL-6, IL-8, and IL-1Ra levels are associated with Cryptococcus infection alone); and (3) the IL-6, GM-CSF, and IL-8 levels are positively correlated with the CSF fungal burden, which is associated with adverse outcomes in HCM patients.

Cryptococcus infection is considered to be an indicator of advanced AIDS stage, which is characterized by a low CD4 cell count. Accordingly, the findings of our study suggest that a lower CD4 count is not only a risk factor for HCM acquisition but is also associated with HICP. That is, HCM patients with ICP ≥ 300 mmH_2_O had a lower CD4 count than those with ICP < 300 mmH_2_O. This finding is in agreement with some previously published studies [18, 19], but it is contradicted by Bicanic et al.'s study [5]. The reason for this is probably that high fungal burden appeared necessary but not sufficient for the development of high pressure [5].

It is widely accepted that cytokines are associated with the immunopathogenesis of HCM [11, 15, 20]. In agreement with this, our study findings show that HCM patients had higher CSF levels of cytokines and chemokines than non-HIV patients and even HIV-infected patients without CNS infection. In particular, HCM patients had increased CSF levels of Th1 cytokines (such as TNF-*α*, TNF-*β*, IFN-*γ*, IL-10, and IL-12), Th2 cytokines (such as IL-4, IL-5, IL-6, and IL-10), and macrophage cytokines (such as IL-8, IL-10 and MCP-1), indicating an unbalanced status of Th1-Th2 cytokine in CSF. The Th1-Th2 cytokine balance in hosts is profoundly associated with the outcome of infection caused by microbes. Th1 cytokines are typically associated with the protective response against Cryptococcus, whereas Th2 cytokines are associated with the ability of the host to effectively control the Cryptococcus infection. However, an exaggerated Th1 response may lead to extensive inflammation and adverse clinical outcomes [21, 22]. In our study, the CSF levels of the Th1 cytokines IL-12 and TNF-*α* were positively associated with both HCM and HICP. Thus, an exaggerated Th1 response may play a pivotal role in CM acquisition as well as HICP in HIV-positive patients.

The Th2 response is considered as a double-edged sword in the pathogenesis of cerebral cryptococcosis, as the Th2 cytokines also activate the release of macrophage cytokines. It has been shown that Th2 cells are dominant in the early stages of Cryptococcus infection [23]. In HIV-infected individuals, a decrease in the percentage of Th1 cells among CD4+ T cells can be detected, while the frequency of Th2 cells is found to be increased [24, 25]. The most important Th2 cytokines are IL-4, IL-6, and IL-10, which are considered to be mediators of macrophage activation. In fact, IL-4-activated macrophages are associated with uncontrolled cryptococcal meningitis [26], and the IL-4 level was reported to decrease after effective treatment of oral candidiasis in HIV-infected patients [27]. In our study, the HCM patients with HICP had higher levels of the Th2 cytokines IL-4, IL-5, IL-6, and IL-10, which might have contributed to macrophage activation. Indeed, macrophage cytokines (such as IL-8) were obviously elevated both in HCM patients and in HCM patients with HICP. Interestingly, our data suggested that elevated but insufficient IL-4 was associated with HICP and higher CSF *Cryptococcus* count. This was reflected in PC3 in Figure 3(b) that IL-4 was negatively overweighed in PC3. Thus, in agreement with the previous study [26], our findings also indicate that IL-4 is associated with uncontrolled, severe CM.

With regard to the Th1-Th2 balance, it has been reported that CD40L activates macrophages to produce immunosuppressive IL-10, and the Th1 cytokine IFN-*γ* optimizes CD40L-induced macrophage activation, thus inducing a switch from IL-10 to IL-12p70 production and promoting macrophage-mediated Th1 T cell skewing [28]. Based on this finding and our present findings, we hypothesized that skewing Th1-Th2 imbalance via CD40L-activated macrophages deteriorated the *Cryptococcus* infection in CNS.

The Cryptococcus-specific CD4+ memory T cell response is associated with the outcomes of HCM patients. A study by Jarvis et al. reported that IFN-*γ* and TNF-*α* production by Cryptococcus-specific CD4+ memory T cells is higher in living patients than in dead patients [29]. Further, higher pretreatment CSF concentrations of IL-4, IL-6, IL-8, IL-10, IL-17, IFN-*γ*, and TNF-*α* are predictive of the risk of early mortality and IRIS in HCM patients [15]. Although HICP and cytokine profiles at admission have been associated with mortality among CM patients [3, 20], we did not observe a direct effect of HICP and baseline CSF cytokine/chemokine profiles on mortality in the present study. This is probably partly because ventriculoperitoneal shunt placement was performed in patients with HICP, which procedure is known to significantly reduce mortality in HCM patients [3], partly because mannitol was used as medication to relieve the HICP in China to reduce mortality [30, 31].

Our present data confirmed the findings of our and others' researchers that HIV-infected CM patients had a significantly lower number of CSF lymphocytes than HIV-negative CM patients [17, 32]. Further, the present findings illustrate that patients with a lower CD4 count in peripheral blood and lymphocyte count in CSF exhibit an uncontrolled increase in their cytokine/chemokine levels and ICP. This is probably because lower lymphocyte counts in peripheral blood and in CSF indicate poor immune status, which probably facilitates Cryptococcus dissemination in the blood and proliferation in CSF.

Although our study presents some pertinent findings, the limitations of our study must also be acknowledged. A small-scale study has indicated that the HIV RNA loads are higher in the CSF of HCM patients than in the CSF of patients without Cryptococcus infection [33]. However, we did not measure the CSF HIV-RNA load in this study, so we were unable to determine whether it was associated with the cytokine/chemokine levels. Another limitation was that the CSF cryptococcal antigen titer was not measured. A previous study has shown that the CSF cryptococcal antigen titer is correlated with CSF fungal burden [34], so it might be meaningful to assess the relationship between CSF cryptococcal antigen titer and CSF cytokine/chemokine. However, we assessed the relationship between CSF fungal count, rather than CSF cryptococcal antigen titer, and CSF cytokine/chemokine concentration. It is reasonable to assess the relationships between CSF cryptococcal antigen titer and CSF cytokine/chemokine among HCM patients in the future.

In summary, the findings of our study show that increased levels of both proinflammatory cytokines (CD40L, IL-12, IL-1*β*, and TNF-*α*) and anti-inflammatory cytokines (IL-1R*α*, TNF-*β*, and INF-*α*) in the CSF played a role in the development of CM and HICP in HIV-infected patients. In particular, the chemokine and cytokine cascade caused by skewing of the Th1-Th2 balance and lower CD4 count together contribute to the development of HICP in HCM patients.

## Figures and Tables

**Table 1:** Patient baseline characteristics (*N* = 82).

Parameter	HIV-Crypt- (*n* = 17)	HIV+Crypt- (*n* = 26)	HIV+Crypt+ (*n* = 39)	*P* value
Age (y) (mean ± SD)	38.0 ± 10.6	36.5 ± 11.9	36.8 ± 11.6	0.761
Sex (male/female)	11/6	22/4	37/2	0.004
CD4+ (cells/*μ*l)	—	82 (18.0-266.0)	35 (15.0-55.3)	0.043
CSF parameter				
Intracranial pressure (mmH_2_O)	151 (133-188)	150 (105-182)	350 (280-450)	<0.001
Lymphocyte count (cells/*μ*l)	1 (0-3.0)	1 (0-2.0)	8 (2.0-20.0)	<0.001
Chlorine (mmol/l)	122.1 ± 6.1	119.3 ± 9.3	119.0 ± 4.6	0.026
Glucose (mmol/l)	3.5 ± 0.7	3.3 ± 0.6	2.8 ± 1.0	0.008
Total protein (g/l)	0.3 (0.2-0.4)	0.3 (0.2-0.4)	0.4 (0.2-0.6)	0.050
Albumin (mg/dl)	20.7 (14.7-26.7)	15.0 (12.8-19.3)	20.4 (13.6-34.1)	0.164
*β*-Microglobulin (mg/dl)	3.8 (2.3-6.6)	4.7 (3.3-6.0)	8.4 (3.9-16.8)	0.006
Cryptococcus (cells/HPF)	—	—	3 (1-30)	—

HIV-Crypt-: patients without HIV or *Cryptococcus* infection; HIV+Crypt-: patients with HIV but without *Cryptococcus* infection; HIV+Crypt+: patients with both HIV and *Cryptococcus* infection; HPF: high-power field (on microscope).

## Data Availability

All data is included in manuscript.
